# SGIV Induced and Exploited Cellular De Novo Fatty Acid Synthesis for Virus Entry and Replication

**DOI:** 10.3390/v14020180

**Published:** 2022-01-18

**Authors:** Qi Zheng, Youhua Huang, Liqun Wang, Ya Zhang, Xixi Guo, Xiaohong Huang, Qiwei Qin

**Affiliations:** 1 Guangdong Laboratory for Lingnan Modern Agriculture, Joint Laboratory of Guangdong Province and Hong Kong Region on Marine Bioresource Conservation and Exploitation, College of Marine Sciences, South China Agricultural University, Guangzhou 510642, China; max_chaos@163.com (Q.Z.); huangyh@scau.edu.cn (Y.H.); Wangliqun1992@163.com (L.W.); yazhang@scau.edu.cn (Y.Z.); guoxixig@126.com (X.G.); 2 Southern Marine Science and Engineering Guangdong Laboratory, Zhuhai 519082, China; 3 Laboratory for Marine Biology and Biotechnology, Qingdao National Laboratory for Marine Science and Technology, Qingdao 266000, China

**Keywords:** SGIV, fatty acid synthesis, ACC1, FASN, virus entry, immune response

## Abstract

Considerable attention has been paid to the roles of lipid metabolism in virus infection due to its regulatory effects on virus replication and host antiviral immune response. However, few literature has focused on whether lipid metabolism is involved in the life cycle of lower vertebrate viruses. Singapore grouper iridovirus (SGIV) is the causative aquatic virus that extensively causes fry and adult groupers death. Here, the potential roles of cellular de novo fatty acid synthesis in SGIV infection was investigated. SGIV infection not only increased the expression levels of key enzymes in fatty acid synthesis in vivo/vitro, including acetyl-Coenzyme A carboxylase alpha (ACC1), fatty acid synthase (FASN), medium-chain acyl-CoA dehydrogenase (MCAD), adipose triglyceride lipase (ATGL), lipoprotein lipase (LPL) and sterol regulatory element-binding protein-1 (SREBP1), but it also induced the formation of lipid droplets (LDs), suggesting that SGIV altered de novo fatty acid synthesis in host cells. Using the inhibitor and specific siRNA of ACC1 and FASN, we found that fatty acid synthesis was essential for SGIV replication, evidenced by their inhibitory effects on CPE progression, viral gene transcription, protein expression and virus production. Moreover, the inhibitor of fatty acid *β*-oxidation could also reduce SGIV replication. Inhibition of fatty acid synthesis but not β-oxidation markedly blocked virus entry during the life cycle of SGIV infection. In addition, we also found that inhibition of ACC1 and FASN increased the IFN immune and inflammatory response during SGIV infection. Together, our data demonstrated that SGIV infection in vitro regulated host lipid metabolism and, in that process, cellular fatty acid synthesis might exert crucial roles during SGIV infection via regulating virus entry and host immune response.

## 1. Introduction

Increased studies have indicated that many viruses could disrupt or rearrange host lipid metabolism, especially the core metabolic pathways, including the metabolism of triglyceride, phospholipid, and cholesterol, to facilitate their virus replication [1,2,3]. Human cytomegalovirus (HCMV) activates glycolysis to supply the tricarboxylic acid cycle during infection, eventually producing fatty acids that are important for infection. Herpesvirus, hepatitis B virus (HBV), Epstein–Barr virus (EBV), human immunodeficiency virus (HIV) and measles virus all disturb or induce fatty acid synthesis [2,4]. In addition, hepatitis C virus (HCV) disrupts different facets of lipid metabolism, alters phospholipid metabolism, and terminates fatty acid transport in mitochondria. West Nile virus (WNV) and HCV virus regulates a key flux-controlling enzyme, cholesterol synthase HMGCR (3-Hydroxy-3-Methylglutaryl-CoA Reductase), to affect the distribution and abnormal content of cholesterol in host cells [5]. It is further proven that virus-mediated regulation of lipid metabolism plays an extensive role in the process of viral infection.

It is well known that lipid metabolism is involved in virus entry, viral genome replication, virions production and assembly [6]. Acetyl CoA carboxylase 1 (ACC1) and fatty acid synthase (FASN) are key nodes in the de novo fatty acid synthesis pathway simultaneously with the lipid metabolism pathway and is crucial against viral infection. For example, ACC1 acts as the first rate-limiting enzyme in the FAs biosynthesis pathway, and the addition of the inhibitor TOFA, an inhibitor of ACC1, can significantly inhibit viral replication [7]. FASN catalyzes the synthesis of long-chain saturated fatty acids from ACC1 in the presence of substrate fatty acids (FAs). In the process of HCV infection, the expression of FASN is upregulated, while the entry and production of viruses are significantly inhibited after treatment with inhibitor C75 or silencing FASN with siRNA [8]. Further studies have shown that Dengue virus (DENV) nonstructural protein 3 specifically recruits FASN to the virus assembly site, a key process for virus replication [9]. Recently, there has been evidence that shows that FAs oxidation also plays an important role in host cells during HBV infection [10].

*Iridoviridae* is a family of viruses with double-stranded DNA genomes. Amphibians, fish, and invertebrates such as arthropods serve as natural hosts. There are currently 22 species in this family, divided among two subfamilies and five genera, including *Ranavirus*, *Megalocytivirus*, *Lymphocystivirus*, *Iridovirus* and *Chloriridovirus* [11]. *Ranavirus* is the only one genus that can infect teleost, amphibians and reptiles in Iridoviridae. The members of *Ranavirus* are serious pathogens to aquatic animal in decade years, and they cause not only the problem of ecological security but also heavy economic losses. Infectious spleen- and kidney-necrotizing virus (ISKNV) is a species of *Megalocytivirus*, and preliminary results have been obtained in the study of the metabolic pattern of ISKNV in vitro [12,13]. Singapore grouper iridovirus (SGIV), a novel species of *Ranavirus*, was isolated from diseased grouper in Singapore. As an enveloped double-stranded large DNA virus, SGIV is the causative aquatic virus that extensively causes fry and adult groupers death, while the mechanisms of SGIV that regulate and exploit host metabolism are limitedly unknown.

In the present study, to clarify the potential roles of cellular de novo fatty acid synthesis in SGIV infection, the transcription level of key enzymes involved in fatty acid synthesis were examined during SGIV infection in vitro or in vivo. Moreover, the roles of key molecules or events related to fatty acid synthesis in SGIV infection were also investigated by specific inhibitors or siRNA targeting ACC1, FASN as well as fatty acid *β*-oxidation.

## 2. Materials and Methods

### 2.1. Cell and Virus

Grouper spleen (GS) cells were established and maintained in our laboratory. GS cells were grown in Leibovitz’s L15 medium containing 10% fetal bovine serum (Gibco) at 25 °C [14]. Propagation of SGIV was performed as described previously [15].

### 2.2. RNA Extraction and Quantitative PCR (qPCR)

To determine the lipid relative genes and virus replication dynamics, the transcription patterns were detected by qPCR. For instance, after pretreatment with acetyl-CoA carboxylase (ACC1) inhibitor C75 (15 μM), FASN inhibitors 5-(tetradecyloxy)-2-furoic acid (TOFA) (30 μM) or fatty acid *β*-oxidation inhibitor etomoxir (15 μM), GS cells were infected with SGIV for different infection times (12, 24 h.p.i.), respectively. Total RNA from mock- or SGIV-infected cells were isolated using the SV total RNA isolation system (Promega) according to the manufacturer’s instruction. The total RNA was detected by electrophoresis on 1% agarose gel and was reverse transcribed using ReverTra Ace qPCR RT Kit (TOYOBO). Amplification was examined using SYBR Green I Reaction Mix (Toyobo) in the ABI QuantStudio 6. Each sample contained three independent individuals collected for RNA extraction and gene expression analysis. The results were calculated based on the expression levels of targeted genes normalized to *β*-actin at the indicated time. The data were represented as mean ± standard deviation (SD). All the primers of target genes were described in the previous study [16]. Statistical significance was determined with Student’s *t* test and the statistical significance was set at *p* < 0.05. The sequences of primers siRNAs and the GenBank accession numbers of genes used in this study are shown in Table 1 and Table 2.

To further analyze the actions of different inhibitors on virus entry, cells were pretreated with inhibitors and infected with virus for 2 h, as described previously [17]. Both the mock- and SGIV-infected cells were collected for further qPCR analysis.

### 2.3. Staining and Quantification of Lipid Droplets

The occurrence of lipid droplets during SGIV infection was evaluated by microscopic examination of cells stained with BODIPY^®^ 493/503 (Invitrogen) as described previously [18]. Mock- or SGIV-infected cells were incubated with 5 μM BODIPY^®^ 493/503 for 15 min and then stained with DAPI. Images were taken under fluorescence microscopy. To measure the quantification of lipid droplets marked by BODIPY^®^ 493/503, we detected a fluorescence signal by flow cytometry [19].

### 2.4. Virus Titer Determination

To investigate the function of fatty acid biosynthesis in virus replication, the siRNA and inhibitor-treated GS cells were harvested to detected virus titers to indicate time points. GS cells were seeded in 96-well plate for 18–24 h and then infected with serial 10-fold dilutions of SGIV samples in 8 replicates. After 96–144 h.p.i., the 50% tissue culture infective dose (TCID_50_) assay was determined using the Spearman–Karber method.

### 2.5. Immunofluorescence Assay

Mock- and SGIV-infected cells at 24 h.p.i. were fixed in methanol at room temperature for 30 min and then permeabilization in 100% ethanol at −20 °C for 10 min. After washing with PBS, the glass-bottom cell culture dishes were blocked by 2% bovine serum albumin for 30 min. Mock- or SGIV-infected cells were incubated with rabbit anti-FASN (1:200) for 2 h and then stained with secondary antibody anti-mouse IgG Fab2 Alexa Fluor 555 (1:100; Molecular probe). The distribution profile of FASN was observed under fluorescence microscopy (Zeiss). To designate the localization of the nucleus, cells were stained with 6-diamidino-2-phenyl-indole (DAPI). The images were taken by microscopy.

### 2.6. Western Blot Assays

GS cells infected with virus were harvested and lysed with RIPA buffer (Pierce). The total protein concentration was measured using a BCA protein assay kit (Kaiji) according to the manufacturer’s instructions. Proteins were separated by 8% or 10% SDS-PAGE and transferred to polyvinylidene difluoride (PVDF) membranes (Millipore). Blots were incubated with anti-SGIV MCP, anti-FASN or anti-*β*-tubulin at a dilution of 1:2000 for 2 h and washed and incubated with horseradish peroxidase (HRP)-conjugated sheep anti-rabbit IgG at a dilution of 1:5000 for 2 h. After washing, protein bands were visualized with an enhanced HRP-DAB Substrate Chromogenic Kit (Tiangen). Signal intensities were quantified using ImageJ software, with *β*-tubulin as an internal reference.

### 2.7. Virus Entry Assay

Whether lipid metabolism affected SGIV entry was investigated using two strategies, namely observation under laser scanning confocal microscope (CLSM, Zeiss) and qPCR. GS cells were seeded in glass-bottom cell culture dishes and pre-treated with C75, TOFA or etomoxir for 2 h. After the indicated times, the cells were prechilled at 4 °C for 5 min and infected with Cy5-labeled SGIV at 4 °C for 20 min in serum-free medium at 28 °C for 1 h. After being washed three times with medium, cells were fixed with 4% paraformaldehyde overnight and stained with the lipophilic dye DiO for 30 min [20]. The cells were observed under CLSM and photographed. In each sample, 30 cells were selected for analysis, and the same protocol was duplicated 3 times; finally, the data were represented as mean ± SEM. The photos were analyzed by the self-compiled program in Matlab (https://www.mathworks.com/products/matlab.html, 11 November 2020) [17,20].

The effects of lipid metabolism on SGIV entry were determined using qPCR. In brief, GS cells were pre-treated with inhibitors for 2 h and infected with SGIV in serum-free medium. After 1 h, cells were washed three times with cold serum-free medium to remove unbound virus. Medium with inhibitors was added, and the cells were cultured for another 4 h at 28 °C. Cells were washed once with medium and treated with 0.2 mL citric acid buffer (citric acid 40 mM, potassium chloride 10mM, sodium chloride 135 mM, pH 3.0) for 1min. Cells were harvested for RNA extraction and qPCR analysis to measure the amount of virus that had entered the cells [21].

### 2.8. Statistical Analysis

The studied data were expressed as means ± SD. Statistical comparison and analysis were performed using SPSS 20 software. Significant differences were indicated with *p* value < 0.05 (Shown as *).

## 3. Results

### 3.1. SGIV Infection Increased the Transcription Level of Key Enzymes Involved in Fatty Acid Synthesis In Vivo/Vitro

**Figure 1:**
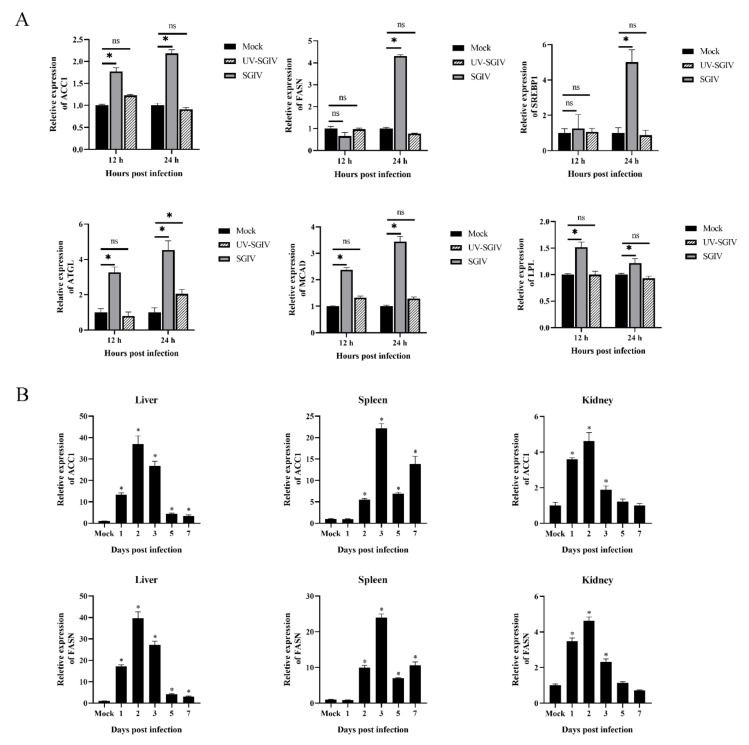
The mRNA expression levels of lipid relative genes post SGIV infected in vivo/vitro. Expression levels of lipid related genes in GS cells during SGIV infection (**A**). mRNA expression of ACC1 and FASN in different tissues of grouper during SGIV infection (**B**). n = 3, data are expressed as means ± SD, * indicates *p* < 0.05, ns for no significant difference.

We first examined the transcription levels of key enzymes involved in fatty acid synthesis in SGIV-infected cells, including ACC1, FASN, SREBP1, ATGL, MCAD and LPL. In vitro, UV-inactivated and wild-type virus-infected GS cells were harvested for qPCR analysis. As shown in Figure 1A, the expression level of genes was increased in SGIV-infected cells with infection time compared to that in mock-infected cells, indicating that SGIV-infection increased fatty acid synthesis in vitro. In contrast, no obvious changes in the expression of these genes were observed in UV-inactivated virus-infected cells, suggesting that upregulation of these key enzymes during SGIV infection might require viral genome replication.

In vivo, the groupers were divided into mock and SGIV-infected groups, and fish were sacrificed at indicate time points. qPCR analysis of SGIV core genes in liver, spleen and kidney, including MCP and VP19 (Figure S1), were performed, suggesting that SGIV replicated well in the infected group. ACC1 and FASN showed similar expression patterns after SGIV infection, and rose significantly versus mock, and then peaked at 2 d in liver and 3 d in spleen and kidney. Besides the peak at 3 d, the expression of two genes were upregulated at 7 d again followed by a valley at 5 d (Figure 1B). In combination with the above experimental results in vitro and in vivo, it is suggested that SGIV infection could increase the transcription level of key enzymes involved in fatty acid synthesis.

### 3.2. SGIV Infection Induced the Formation of Lipid Droplets (LDs)

**Figure 2:**
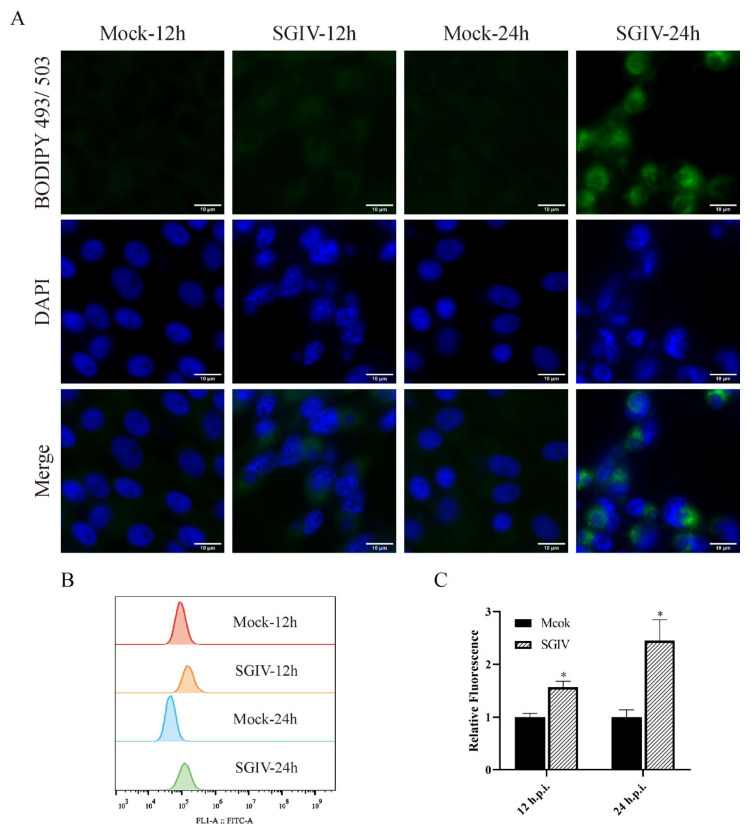
SGIV infection induced the formation of lipid droplets. The lipid droplets in SGIV-infected cells were stained with BODIPY^®^ 493/503. Mock or SGIV-infected cells were stained with BODIPY^®^ 493/503 for 15 min and then observed under fluorescence microscopy (**A**). The quantity of LDs was measured by flow cytometry, and the 4 curves indicate the fluorescence intensity (**B**,**C**). n = 3, data are expressed as means ± SD, * indicates *p* < 0.05.

It has been demonstrated that many viruses can induce the formation of LDs that are beneficial for virus replication [22]. To determine whether the formation of LDs occurred in SGIV-infected cells, mock or infected cells were stained with BODIPY^®^ 493/503, and the cells were observed by fluorescent microscope. As shown in Figure 2A, the detectable green fluorescent signals were increased in SGIV-infected cells (Figure 2A). In addition, they were quantitatively analyzed by flow cytometry, the relative fluorescence of infectious cells was 1.57 and 2.45 times more than that in mock infection cells at 12 and 24 h.p.i., respectively. These data suggest that SGIV infection can induce the formation of LDs.

### 3.3. Fatty Acid Synthesis Was Essential for SGIV Replication

**Figure 3:**
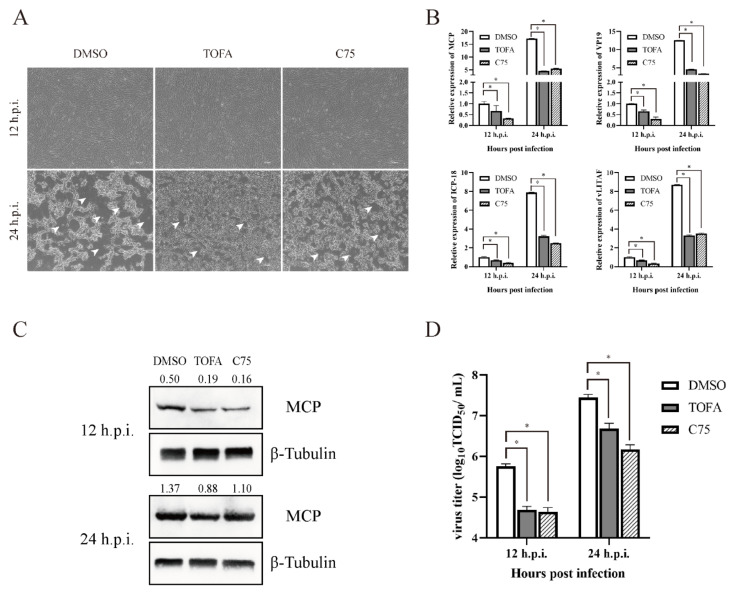
Roles of fatty acid synthesis during SGIV replication. CPE of TOFA and C75 on GS cells during SGIV infection (**A**). Effects of TOFA and C75 on SGIV genes transcription during SGIV infection (**B**). Effects of TOFA and C75 on SGIV MCP protein expression during SGIV infection (**C**). Effects of TOFA and C75 on SGIV TCID_50_ during SGIV infection (**D**). n = 3, data are expressed as means ± SD, * indicates *p* < 0.05.

Some viruses have been reported that they can manipulate the fatty acid synthesis pathway to increase the replicate efficient [5]. To assess whether fatty acid synthesis is involved in SGIV replication, we focused on ACC1 and FASN, the two most crucial restriction enzymes, and explored their roles in SGIV infection by corresponding inhibitors and specific siRNA. The concentration of inhibitors was used as described in our previous study [22]. GS cells were pretreated with TOFA and C75 for 2 h before SGIV infection, and a cytopathic effect (CPE) could be observed at 12 h post infection (h.p.i.). The CPE showed significant delays in TOFA- and C75-treated cells than in DMSO-treated cells at 24 h.p.i. (Figure 3A). Consistently, the transcription of viral genes, including MCP, VP19, ICP-18 and vLITAF, were all significantly decreased in SGIV-infected cells in the presence of TOFA or C75 (Figure 3B). The expression levels of MCP and virus production were significantly suppressed after FAs synthesis inhibitor (TOFA and C75) treatment (Figure 3C,D).

**Figure 4:**
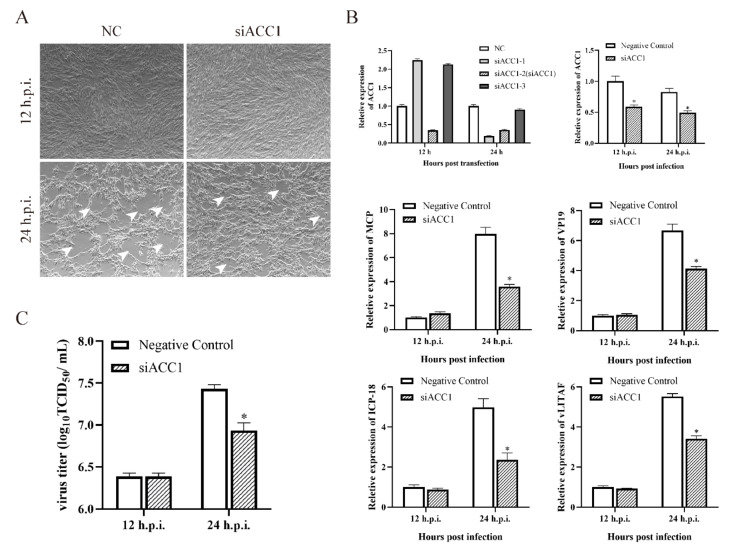
Roles of ACC1 during SGIV replication. CPE of siRNA on GS cells during SGIV infection (**A**). Selection of siRNA, and the most effective siRNA of ACC1 suppress SGIV genes transcription during SGIV infection (**B**). Effects of siRNA on SGIV TCID_50_ during SGIV infection (**C**). n = 3, data are expressed as means ± SD, * indicates *p* < 0.05.

To clarify the role of ACC1 in SGIV infection, three different candidate siRNAs were designed to silence the transcription of ACC1. The siACC1-2 showed the most significant silencing effect on ACC1 expression and was designated as siACC1 in the following experiments (Figure 4B). As shown in Figure 4A, the severity of CPE induced by SGIV was obviously weakened by transfection with siACC1. Consistently, the viral gene transcription and virus production in siACC1-transfected cells were also significantly inhibited at 24 h.p.i. compared with control cells (Figure 4B,C).

**Figure 5:**
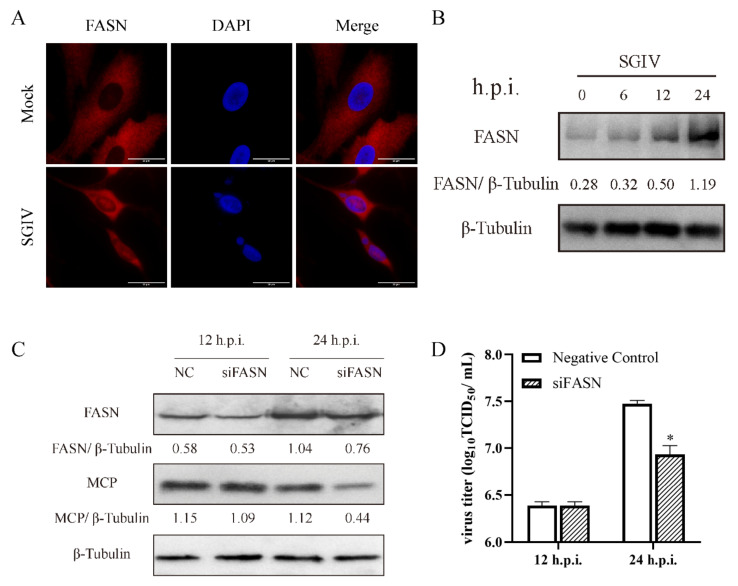
Roles of FASN during SGIV replication. Immune fluorescence detection of the FASN protein after treatment with mock and SGIV. Red fluorescence indicates FASN protein (**A**). Protein expression of FASN during SGIV infection (**B**). Effects of siRNA on SGIV MCP protein expression during SGIV infection (**C**). Effects of siRNA on SGIV TCID_50_ during SGIV infection (**D**). n = 3, data are expressed as means ± SD, * indicates *p* < 0.05.

As the direct enzyme of fatty acid synthesis, it is crucial to determine the role of FASN [23]. Subcellular localization of FASN was labeled by antibodies. The immunofluorescence microscopy observation showed that FASN was distributed in cytoplasm in mock and SGIV-infected cells at 24 h.p.i., while the fluorescence intensity of FASN was enhanced in SGIV-infected cells (Figure 5A). Results of the FASN protein expression pattern also supported the results obtained from immunofluorescence microscopy (Figure 5B). To further determine if FASN was involved in SGIV replication, we knocked down FASN in GS cells by using siRNA as described in a previous study [22]. As shown in Figure 5C, when FASN was silenced, the level of SGIV MCP protein was also reduced versus negative control (NC), besides that the virus titer was 10^7.48^ TCID_50_/mL in NC cells and then decreased to 10^6.93^ TCID_50_/mL in FASN-interfered cells (Figure 5D). These results implied that de novo fatty acid biosynthesis is involved in SGIV infection and that FASN and ACC1 are essential for SGIV replication.

### 3.4. Inhibition of Fatty Acid β-Oxidation by Etomoxir Decreased SGIV Replication

**Figure 6:**
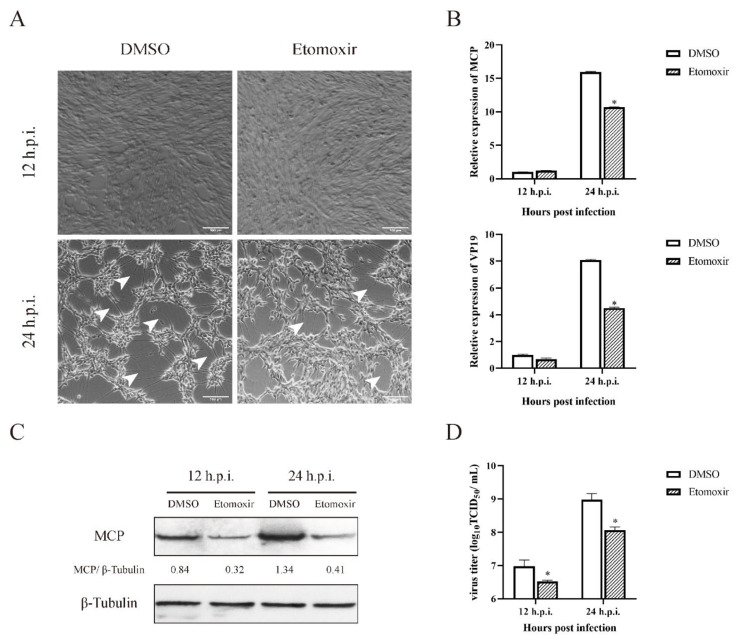
Roles of fatty acid *β*-oxidation during SGIV replication. CPE of GS cells under etomoxir during SGIV infection (**A**). Effects of etomoxir on SGIV genes transcription during SGIV infection (**B**). Effects of etomoxir on SGIV MCP protein expression during SGIV infection (**C**). Effects of Etomoxir on SGIV TCID_50_ during SGIV infection (**D**). n = 3, data are expressed as means ± SD. * indicates *p* < 0.05.

Growing evidence has demonstrated that *β*-oxidation exerts crucial roles during the virus life cycle. To clarify whether *β*-oxidation was critical for SGIV infection, GS cells were treated by etomoxir, an inhibitor of fatty acid *β*-oxidation, and were then infected with SGIV. As shown in Figure 6A, the CPE induced by SGIV infection was significantly reduced in etomoxir-treated cells compared with to that in DMSO-treated cells at 24 h.p.i. Moreover, viral gene transcription and protein synthesis were both significantly suppressed in etomoxir-treated cells versus DMSO-treated cells (Figure 6B,C). The results of the virus titer assay showed that the mean value of TCID_50_/mL was 10^6.98^ (DMSO 12 h.p.i.), 10^6.53^ (etomoxir 12 h.p.i.), 10^8.98^ (DMSO 24 h.p.i.) and 10^8.07^ (etomoxir 24 h.p.i.). Compared to the control group, the virus production in etomoxir-treated cells decreased up to 35.48% (12 h.p.i.) and 12.30% (24 h.p.i.), revealing that fatty acid *β*-oxidation could also decrease the virus yield (Figure 6D). These data revealed that fatty acid *β*-oxidation is a crucial event during SGIV replication.

### 3.5. Fatty Acid Biosynthesis, but Not β-Oxidation, Participated in the Process of SGIV Entry

**Figure 7:**
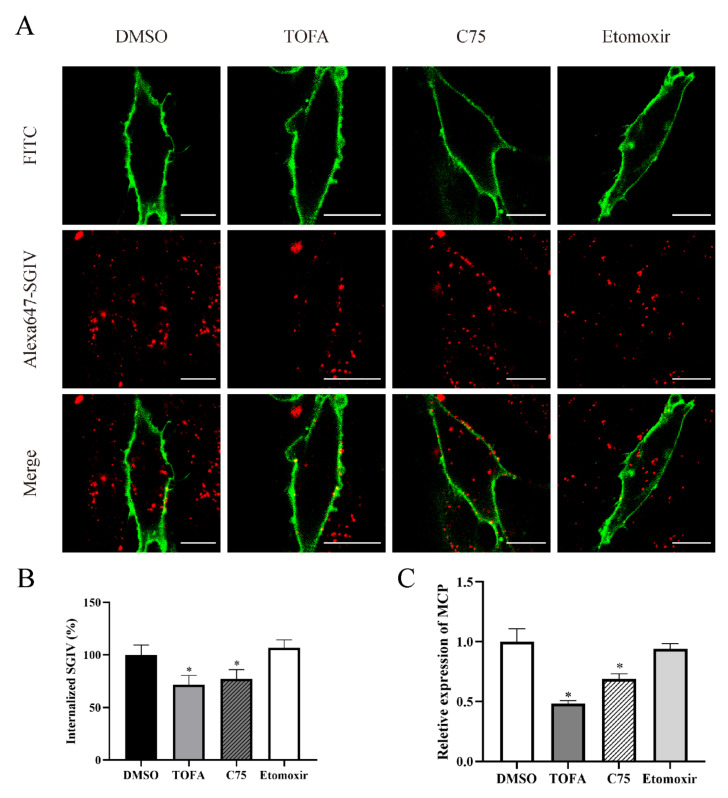
Fatty acid biosynthesis, but not *β*-oxidation, participates in the process of SGIV entry. TOFA and C75 inhibited SGIV entry, and the fixed cells were observed by CLSM (**A**,**B**). The mRNA relative expression of SGIV MCP was suppressed by TOFA and C75 intracellularly (**C**). n = 3, data are expressed as means ± SD. * indicates *p* < 0.05.

To further investigate the role of fatty acid synthesis in the virus life cycle, GS cells were pre-treated with DMSO, TOFA, C75 and etomoxir, and virus entry was evaluated by single-virus tracking. In TOFA- and C75-treated cells, Cy5-labeled SGIV particles in the cytoplasm were significantly reduced to 71.75% and 77.06% compared to DMSO-treated cells, respectively (Figure 7A,B). Otherwise, no significant difference was observed between DMSO- and etomoxir-treated cells. Using qRT-PCR assay, we found that the expression level of SGIV MCP was downregulated to 52% and 32% under the treatment with TOFA and C75, respectively, but no significant difference was observed in SGIV-infected etomoxir-treated cells compared to DMSO-treated cells (Figure 7C). Thus, our results revealed that fatty acid biosynthesis, but not *β*-oxidation, participates in the process of SGIV entry.

### 3.6. Inhibition of De Novo Fatty Acid Biosynthesis Increased the IFN Immune and Inflammatory Response

**Figure 8:**
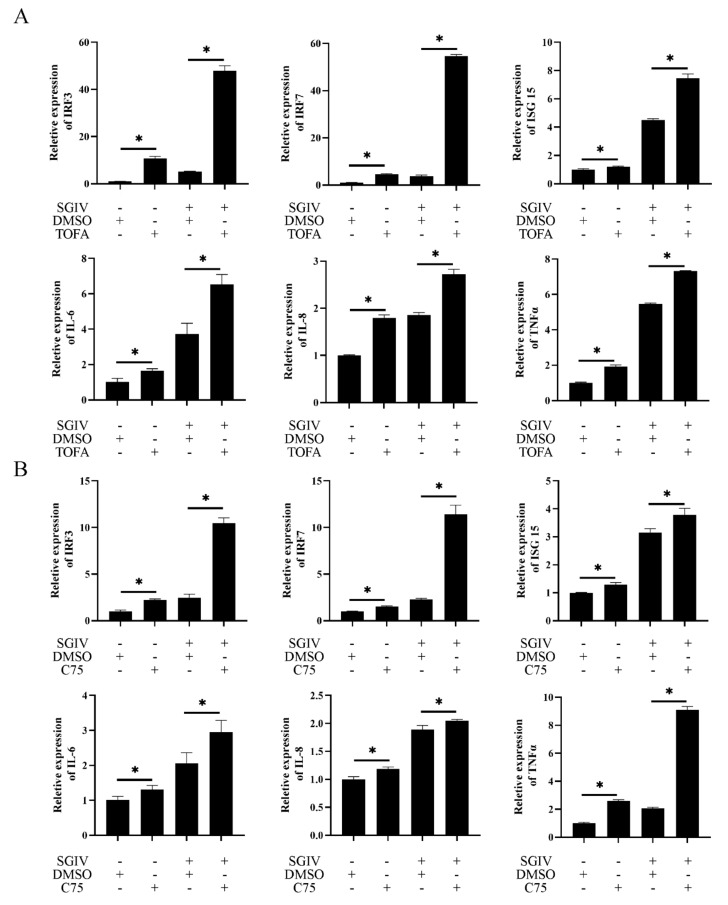
Inhibition of de novo fatty acid biosynthesis increases the IFN immune and inflammatory response. GS cells were preincubated with DMSO, TOFA and C75, and then infected with Mock/SGIV. The IFN-related genes, including IRF3, IRF7 and ISG15, were markedly upregulated (**A**), including in proinflammatory factors such as IL-6, IL-8 and TNFα (**B**). n = 3, data are expressed as means ± SD. * indicates *p* < 0.05.

To examine the roles of fatty acid biosynthesis on host immune response against SGIV infection, GS cells were pre-incubated with TOFA or C75 and then infected by Mock/SGIV. As shown in Figure 8, the expression levels of IFN-related genes, including IRF3, IRF7 and ISG15, were significantly increased during FA synthesis inhibition (Figure 8A). Similarly, the expression levels of proinflammatory cytokines, such as IL-6, IL-8 and TNFα, were also upregulated (Figure 8B). Thus, we speculated that fatty acids exerted an essential role in host immune and proinflammatory responses during SGIV infection.

## 4. Discussion

Recent studies have demonstrated that viruses are able to induce the rearrangement of lipid metabolism and exploit the cellular lipids to facilitate viral multiplication during infection [24,25,26]. In our study, the transcription levels of several key enzyme genes involved in FA synthesis were significantly increased during the infection of wild-type SGIV in vitro, but not UV-inactivated viruses, suggesting that the alteration of lipid metabolism in host cells was dependent on SGIV replication. Consistently, the mRNA expression levels of ACC1 and FASN were both significantly upregulated in the liver, spleen and kidney in response to SGIV infection, compared with mock-infected grouper. Different from the liver and kidney, the expression levels of ACC1 and FASN reached peaks in the spleen at 3 and 7 d.p.i. but dropped at 5 d.p.i. Similar phenomena were also observed in other pathogen-infected fish, such as *Oreochromis niloticus* [27], *Carassius auratus* [28] and *Ictalurus punctatus* [29]. The detailed mechanism underlying the expression changes of these enzyme genes against SGIV infection was needed further investigation. LDs are organelles that play a key role in lipid metabolism, and they can be stained with BODIPY^®^ 493/503 in vitro. Using this specific LDs-target dye, we found that the fluorescence signaling was enhanced during SGIV infection and was distributed around the virus assembly site. The alteration of LDs during virus infection was also observed in Brome mosaic virus, Dengue virus, and RGNNV-infected cells [4,22,30,31], suggesting that viruses might recruit cellular components such as lipids and enzymes involved in cell metabolism to benefit their life cycle.

LDs are lipid-rich cellular organelles that regulate the storage and hydrolysis of neutral lipids and are found largely in adipose tissue [32]. A change in LD status means that viruses affect the lipid metabolism of host cells. To elucidate the potential mechanism of fatty acid biosynthesis during SGIV infection, specific inhibitors and siRNAs of FA synthesis enzyme genes, such as ACC1 and FASN (no homologs of these genes in the SGIV genome), were used in this study. Our results indicated that SGIV-evoked CPE progression was significantly weakened along with viral gene transcription, protein synthesis, and virus production upon the inhibition of FA synthesis in vitro. The replication of HCMV, Influenza, DENV and HCV can also be reduced after the inhibition of an FA-synthesis pathway [26,33,34]. In addition, accumulation of fatty acids supplied an ideal environment for virus replication. Does *β*-oxidation of fatty acids also provide a crucial role during SGIV infection? After treating with etomoxir, we found that the replication of SGIV was also significantly decreased compared with the control cells, suggesting that an increase in *β*-oxidation and ATP production might stimulate SGIV replication similar to DENV [4]. A similar phenomenon was also observed during RGNNV infection in vitro [22,35]. Thus, our data suggested that FA synthesis was activated after SGIV infection, and the increasing stock of intracellular FAs might provide an excellent microenvironment for viral replication.

FAs play a marked role in the cell membrane system [36]. It is convincing that inhibition of fatty acid synthesis affects cell membrane composition and virus entry. For example, the foot-and-mouth disease virus (FMDV) and human rhinovirus type 2 entered into cells by clathrin-mediated endocytosis, which requires an efficient internalization of plasma membrane cholesterol [37,38]. Using single virion tracking technology, we found that SGIV entry was severely decreased after the inhibition of the de novo FA synthesis. Given that SGIV entry into host cells via both clathrin-mediated endocytosis and micropinocytosis [20], we speculated that SGIV infection altered the cell membrane composition via changes to the cellular FAs, thus affecting the efficiency of virus entry.

To clarify the effects of de novo FA synthesis on host antiviral immune response, the expression of IFN-signaling molecules and proinflammatory cytokines in Mock/SGIV-infected cells after treatment with FA inhibitors was examined. The expression level of several IFN-related cytokines, including IRF3, IRF7 and ISG15, as well as that of proinflammatory cytokines, such as IL-6, IL-8 and TNFα, were all significantly increased. Increased research has demonstrated that inhibition of the fatty acid synthesis pathway substantially increases the basal expression of antiviral genes via the spontaneous production of type I interferons (IFN) [39]. The lack of ω-3 FAs could disturb lipid metabolism homeostasis and thus induced proinflammation metabolites synthesis [40]. During classical swine fever virus (CSFV) infection, inhibition of FA synthesis enhanced the interferon response and affected virus replication [41]. Our previous study indicated that overexpression of IRF7 significantly inhibited SGIV replication in vitro [16,42]. Thus, we speculated that inhibition of FA synthesis may also affect virus replication via the enhanced antiviral activity of the interferon-related cytokines or proinflammatory factors during SGIV replication.

**Figure 9:**
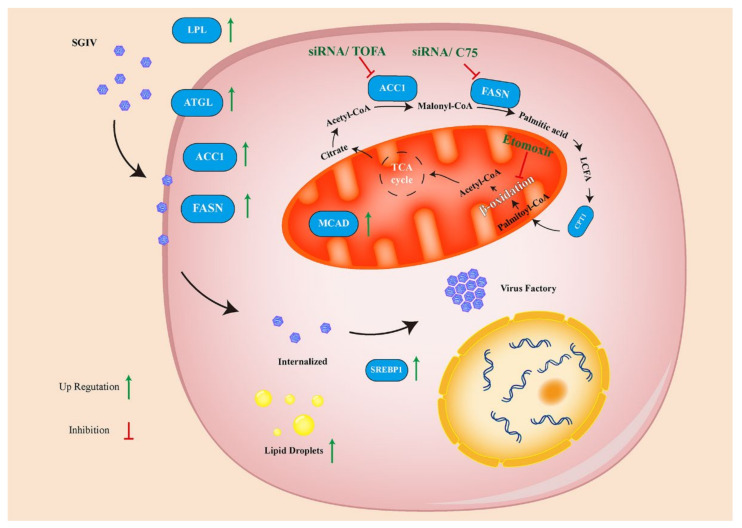
Model for the roles of de novo fatty acid biosynthesis during SGIV infection. The de novo fatty acid biosynthetic pathway was utilized through SGIV infection. Inhibition of ACC1 (converts acetyl-CoA to malonyl-CoA), FASN (catalysis malonyl CoA with acetyl-CoA to generate palmitate) and fatty acid *β*-oxidation significantly decreased SGIV replication. Key FAs synthesis enzymes and processes are shown in blue. Inhibitors and siRNA are shown in dark green.

In summary, we first demonstrated the potential roles of host FA synthesis on SGIV infection in vitro. We found that SGIV infection upregulated the expression level of host key enzyme genes involved in FA synthesis and increased lipid droplets as well as the infection time. Moreover, the disruption of de novo FA synthesis not only decreased virus entry and replication, but also upregulated the expression levels of IFN-related and pro-inflammatory factors (Figure 9). Thus, these results demonstrated that FA synthesis was essential for SGIV infection that may be due to its regulatory effect on virus entry or host interferon immune and inflammatory responses. Together, our findings will provide new insights into understanding iridovirus–host interactions.

## Figures and Tables

**Table 1:** Sequences of primers used in this study.

Primers	Sequences (5′-3′)
RT-ACC1-F	GAAAGGGCAATCCGTTTTG
RT-ACC1-R	GGCGTAGTTGTTGTTATTGGTCC
RT-FASN-F	AAGTCGTTGACCAGCCTATTCCC
RT-FASN-R	TTCACTGCGTCCTCTGTCCGT
RT-MCAD-F	AATACTTGGGAAGGTTGACCGAG
RT-MCAD-R	CTTGCTGGCTGGACATTTAGGA
RT-ATGL-F	TCATTGAGCACCTTCCACCCA
RT-ATGL-R	CGACTTTTAGTAACTGCTCCCGAAT
RT-LPL-F	AAGATGTACCTGAAGACTCGTGAAGTG
RT-LPL-R	TGGTGATAAGGAAGGACAAAGTGG
RT-SREBP1-F	TCATGGTTGGCACGGTGATCT
RT-SREBP1-R	TGCTGAGGGTGCTGGTAGGAT
RT-*β*-actin-F	TACGAGCTGCCTGACGGACA
RT-*β*-actin-R	GGCTGTGATCTCCTTCTGCA
RT-MCP-F	GCACGCTTCTCTCACCTTCA
RT-MCP-R	AACGGCAACGGGAGCACTA
RT-VP19-F	TCCAAGGGAGAAACTGTAAG
RT-VP19-R	GGGGTAAGCGTGAAGACT
RT-ICP-18-F	ATCGGATCTACGTGGTTGG
RT-ICP-18-R	CCGTCGTCGGTGTCTATTC
RT-vLITAF-F	GATGCTGCCGTGTGAACTG
RT-vLITAF-R	GCACATCCTTGGTGGTGTTG
Negative Control	TTCTCCGAACGTGTCACGTTT
siACC1-1	GCGGCATTCAAATCATGCATT
siACC1-2	GCTCCTTCATGGAGATCATTT
siACC1-3	GCUATTAAGGACCTGAACATT
siFASN	GCAAACCACTCTGGTACATTT

**Table 2:** GenBank accession numbers of genes used in this study.

Gene	Accession No.
acetyl-Coenzyme A carboxylase alpha	FJ196229.1
fatty acid synthase	XM_033612145.1
medium-chain acyl-CoA dehydrogenase	XM_033651417
adipose triglyceride lipase	KY649281.1
lipoprotein lipase	EU683732.1
sterol regulatory element-binding protein-1	KT937284.1

## Data Availability

All datasets presented in this study are included in the article.

## Supplementary Materials

The following are available online at https://www.mdpi.com/article/10.3390/v14020180/s1, Figure S1: The mRNA expression of MCP and VP19 in different tissues of grouper during SGIV infection. n = 3, data are expressed as means ± SD. * indicates *p* < 0.05.
