# Supplementary material for: SGIV Induced and Exploited Cellular De Novo Fatty Acid Synthesis for Virus Entry and Replication
Source: Viruses. 2022 Jan 18;14(2):180. doi: 10.3390/v14020180 (PMC8878837; doi:10.3390/v14020180)

viruses

MDPI

Supplementary material

Figure S1. The mRNA expression of MCP and VP19 in different tissues of grouper during SGIV infection. n = 3, data are expressed as means ± SD. * indicates $p < 0.05$.

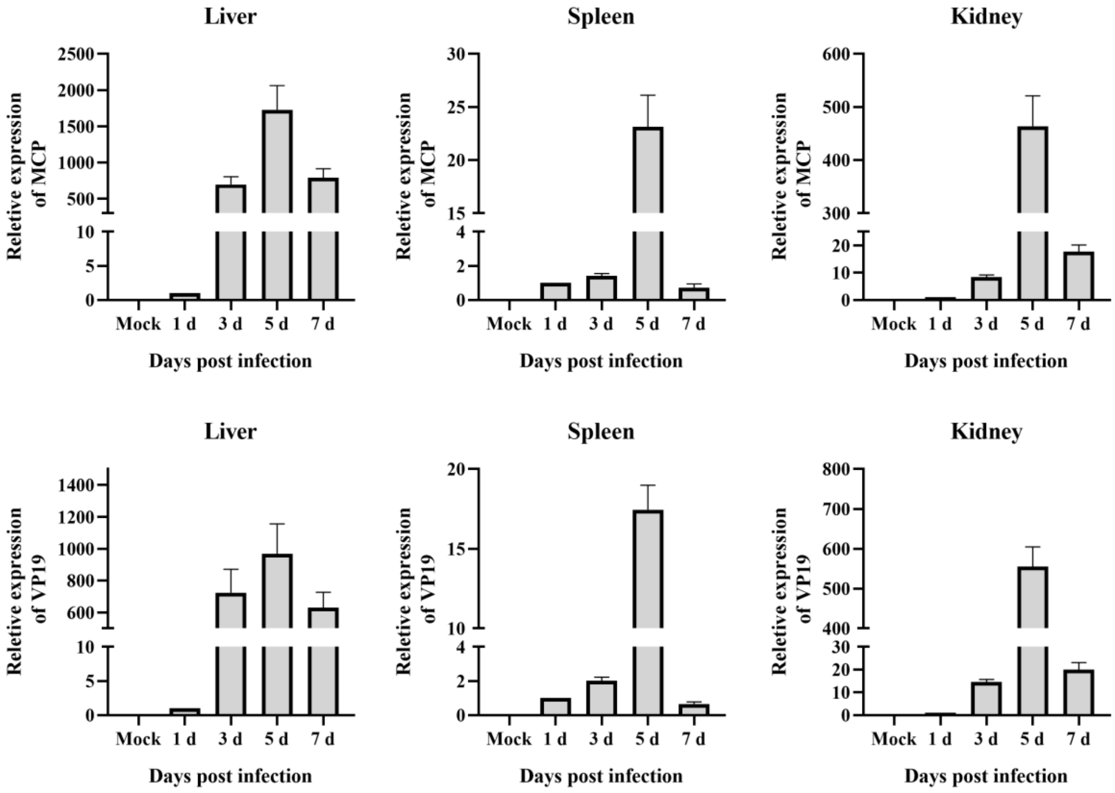

Supplement: Supplementary file 1 [file viruses-14-00180-s001.zip › viruses-1524487-supplementary.pdf]
